# Synthesis, Structure and Performance of an Insensitive Diazonium Inner Salt Energetic Material

**DOI:** 10.3390/molecules31132340

**Published:** 2026-07-03

**Authors:** Haifeng Wang, Jinxin Wang, Ruibing Lv, Yapeng Yao, Pengzhao Han, Wenquan Zhang, Kangcai Wang

**Affiliations:** 1 National Key Laboratory of Chemical Explosion Safety, Institute of Chemical Materials, China Academy of Engineering Physics (CAEP), Mianyang 621900, China; wanghaifeng24@gscaep.ac.cn (H.W.); wangjinxin@casc42.cn (J.W.); lvruibing0719@163.com (R.L.); yaoyp21@163.com (Y.Y.); hpzsdbrk12138@163.com (P.H.); zhangwq-cn@caep.cn (W.Z.); 2 National Key Laboratory of Aerospace Chemical Power, Xiangyang 441003, China

**Keywords:** diazonium, energetic materials, fused-ring, inner salt

## Abstract

Herein, a novel insensitive diazonium inner salt of 2-nitro-5-oxo[1,2,4]triazolo[1,5-c]pyrimidin-8-diazonium-7-olate (**NTPD**) was synthesized through a concise three-step route. The structure and performance of this material were comprehensively studied. Single-crystal X-ray diffraction analysis revealed that **NTPD** possesses a distinctive fused-ring framework featuring an inner salt (zwitterionic) structure, wherein the diazonium and phenolate functionalities are intramolecularly integrated within a compact, highly conjugated heterocyclic system. Furthermore, in comparison with previously reported diazonium compounds, **NTPD** exhibits a superior combination of enhanced thermal stability and significantly reduced mechanical sensitivity. Specifically, its onset decomposition temperature reaches 206 °C, representing a substantial improvement over conventional diazo derivatives, while its impact sensitivity of 7 J positions it among the least sensitive diazonium-based energetic materials reported to date. The exceptional performance of **NTPD** is strongly attributed to its nearly planar molecular geometry and the extensive hydrogen-bonding network present within its crystal lattice, which collectively reinforce structural rigidity, enhance packing stability, and effectively dissipate external mechanical stimuli.

## 1. Introduction

The development of high-performance energetic materials has garnered sustained attention due to their extensive applications in aerospace technology and national defense [1,2]. Energetic materials are typically classified into three categories based on their sensitivities: primary explosives, main (secondary) explosives, and insensitive explosives [3,4]. Diazonium compounds have long been recognized for their inherently high sensitivity, making them well-suited as primary explosives such as diazodinitrophenol (DDNP), a well-known primary explosive [5,6,7]. The inherently high sensitivity of diazonium compounds poses significant safety hazards throughout their entire life cycle, encompassing synthesis, processing, storage, transportation, and end-use applications [8,9]. Therefore, the development of novel diazonium compounds with reduced sensitivity is of paramount importance for advancing the field of safer primary explosives.

**Figure 1:**
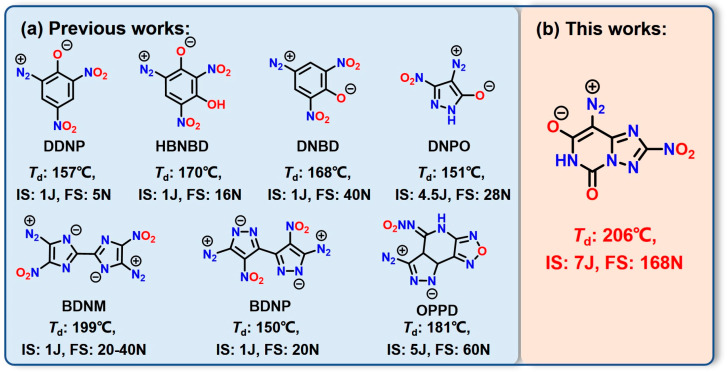
Structures and properties of diazonium inner salt molecules.

In order to reduce the sensitivity of primary explosives, substantial research efforts have been devoted, leading to the design and preparation of numerous energetic materials based on the diazonium inner salt structure (Figure 1a) [7,10,11,12,13,14]. For example, to reduce the sensitivity of DDNP, a hydroxyl group was introduced into 4-hydroxy-3,5-dinitrobenzenediazonium-2-olate (HBNBD) to form hydrogen bonding networks. Compared with DDNP (*T*_d_ = 157 °C, FS: 5 N), HBNBD (*T*_d_ = 170 °C, FS: 16 N) exhibits both improved thermal stability and reduced friction sensitivity. Notably, 3,5-dinitrobenzenediazonium-4-olate (DNBD), an isomer of DDNP, also shows improved thermal stability relative to DDNP, with a decomposition temperature of 168 °C and further reduced friction sensitivity (FS: 40N) [12]. Another representative example is 5,5′-bis(diazonio)-4,4′-dinitro-1,1′-diido-2,2′-biimidazole (BDNM), which consists of two imidazole rings linked by a C–C bond and features an inner bis(diazonium) zwitterionic structure. BDNM exhibits a high decomposition temperature of 199 °C, whereas its isomer 5,5′-bis(diazonio)-4,4′-dinitro-3,3′-bipyrazole-1,1′-diide (BDNP) shows markedly lower thermal stability (*T*_d_ = 150 °C) [13]. Furthermore, the first fused diazonium-based primary explosive, (*E*)-*N*-(6-amino-[1,2,5]oxadiazolo[3,4-b]pyrazolo[5,4-d]pyridin-5(4H)-ylidene)nitramide (OPPD), which maintains reasonable thermal stability (*T*_d_ = 181 °C) and exhibits improved mechanical sensitivities (IS: 5 J, FS: 60 N) [14]. Although the majority of these reported materials still suffer from high sensitivity and inadequate thermal stability, which significantly limit their practical applicability, representative structural modification strategies have demonstrated the possibility of enhancing their safety and stability.

In recent years, nitrogen-rich fused-ring compounds have garnered considerable attention in the field of energetic materials owing to their diverse structures and exceptional performance [15,16,17]. Fused-ring frameworks usually possess rigid, planar, and conjugated skeletons [17,18]. These structural features reduce conformational flexibility and, when suitable hydrogen-bond donors and acceptors are present, favor the formation of directional hydrogen-bonding networks [19,20]. Coupled with the molecular planarity, these intermolecular interactions promote compact and ordered crystal packing, inducing layered packing [20,21]. In layered crystals, molecular-layer slippage under mechanical stimuli can dissipate external energy and relieve local stress, thereby reducing mechanical sensitivity [21,22]. Moreover, dense crystal packing and strong intermolecular interactions, especially hydrogen-bonding networks, can increase lattice energy and stabilize the crystal framework, thereby helping to retard thermal decomposition and improve thermal stability [23]. For example, the fused tricyclic energetic molecule 2,6-diamino-9-iminio-3,5-dinitro-9H-dipyrazolo[1,5-a:5′,1′-d][1,3,5]triazin-4-ide (ZDPT) [24] which we previously obtained, and the fused bicyclic molecule 2-nitro-[1,2,4]triazolo[1,5-a][1,3,5]triazine-5,7-diamine (NTTD) [25] exhibit low sensitivities (IS > 40 J, FS > 360 N) as a result of their planar conjugated frameworks and hydrogen-bonding interactions. Therefore, the development of fused-ring-based diazonium inner salts represents a promising and effective strategy for reducing mechanical sensitivity while maintaining desirable energetic performance.

Continuing our previous efforts toward the development of high-performance energetic materials, herein, a new fused-ring-based diazonium inner salt of 2-nitro-5-oxo[1,2,4]triazolo[1,5-c]pyrimidin-8-diazonium-7-olate (**NTPD**) with excellent sensitivity and great thermal stability was prepared (Figure 1b). The structure and performance of these materials were comprehensively studied by single-crystal X-ray diffraction, infrared spectroscopy (IR), elemental analysis (EA), thermogravimetric analysis (TG), differential scanning calorimetry (DSC), and a series of performance evaluations. In addition, theoretical calculations were carried out to probe the relationship between structural features and molecular stability, while the detonation performance and sensitivity of this energetic compound were systematically assessed. 

## 2. Results and Discussion

### 2.1. Synthesis 

**Scheme 1:**
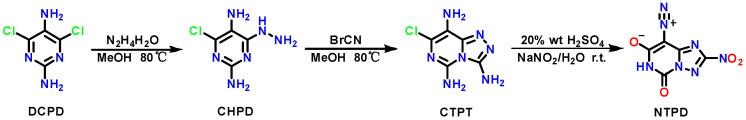
The synthesis route of CHPD, CTPT and **NTPD**.

The synthetic route of **NTPD** is shown in Scheme 1. As shown in Scheme 1, **NTPD** was synthesized through a three-step reaction sequence employing 4,6-dichloropyrimidine-2,5-diamine (DCPD) as the starting material. First, DCPD was refluxed in methanol with hydrazine hydrate at 80 °C to obtain 4-chloro-6-hydrazineylpyrimidine-2,5-diamine (CHPD) [26]. Then, CHPD reacted with BrCN in methanol at 80 °C to form a fused-ring-based compound of 7-chloro-[1,2,4]triazolo[4,3-c]pyrimidine-3,5,8-triamine (CTPT). Subsequent oxidation of CTPT with aqueous sodium nitrite in 20 wt% dilute sulfuric acid afforded 2-nitro-5-oxo[1,2,4]triazolo[1,5-c]pyrimidin-8-diazonium-7-olate (**NTPD**). Notably, a plausible formation pathway for NTPD may involve acid-promoted hydrolysis, diazotization, tautomerization, nitrite-mediated nitration, and subsequent Dimroth rearrangement to give the thermodynamically more stable product (Figure S13). Dimroth rearrangement was also observed in the preparation of other energetic materials of 6-azido-3-nitro-[1,2,3]triazolo[1,5-b][1,2,4]triazin-5-amine [27]. The structure and purity of DCPD, CHPD and **NTPD** were measured by NMR, IR and EA. In addition, mass spectra were obtained for CHPD and NTPD, and the structure of NTPD was further confirmed by single-crystal X-ray diffraction (SXRD).

### 2.2. X-Ray Structure Analysis

**Figure 2:**
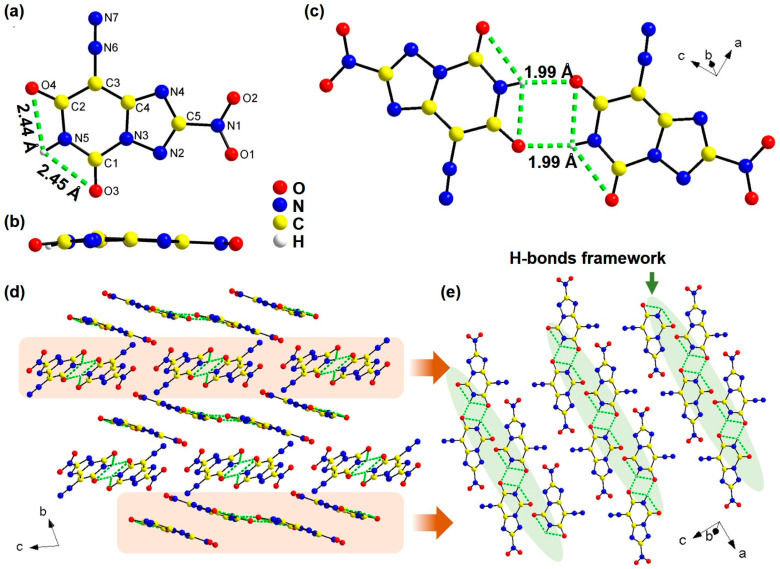
(**a**) The molecular structure of **NTPD**. (**b**) The side view of the molecular structure of **NTPD**. (**c**) The **NTPD** dimer contained in the crystal. (**d**) The crystal stacking of **NTPD** viewed from *a* axis. (**e**) Hydrogen bonding network of **NTPD**.

Single crystals of **NTPD** suitable for X-ray diffraction analysis were obtained from ethyl acetate by slow evaporation. **NTPD** crystallizes in the triclinic space group *P*-1, with a crystal density of 1.79 g cm^−3^ at 292 K. As shown in Figure 2a,b, the molecular skeleton of **NTPD** consists of a fused triazole–pyrimidine ring and shows high planarity; all the atoms of **NTPD** were almost in-plane, with a maximum torsion angle of only 11.3(2)° (O1-N1-C5-N2, Table S3), indicating that the skeleton is highly rigid and essentially coplanar. In addition, two moderately strong intramolecular N–H···O hydrogen bonds are observed, with N···O distances of 2.44 and 2.45 Å, respectively. By comparison, a stronger intermolecular N-H···O hydrogen bond is observed between neighboring molecules, with a corresponding H···O distance of 1.99 Å, linking two molecules of **NTPD** into a dimer (Figure 2c). This result shows that intermolecular hydrogen bonding is stronger than intramolecular hydrogen bonding, which is an important feature of the crystal structure of **NTPD**. In the crystal packing of **NTPD**, the molecules adopt a cross-stacked arrangement along an axis (Figure 2d), while the dimers are further linked by extensive intralayer hydrogen-bonding interactions to form an extended hydrogen-bonded network (Figure 2e). The combined effects of a rigid molecular skeleton, strong intermolecular hydrogen bonding, and cross-stacked packing contribute to the high thermal stability and low mechanical sensitivity of **NTPD**.

### 2.3. Stability 

**Figure 3:**
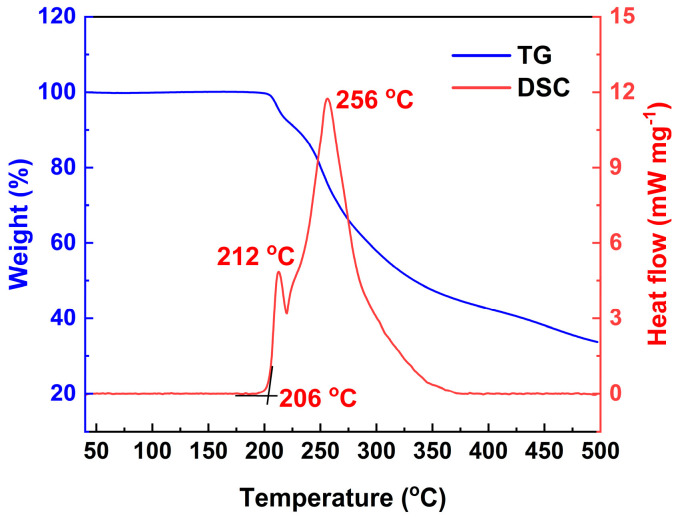
TG and DSC curves of **NTPD**.

Thermal stability is a prerequisite for the safe handling and practical application of energetic materials. The thermal decomposition behavior of **NTPD** was investigated by simultaneous thermogravimetric analysis and differential scanning calorimetry (TG-DSC) under an argon atmosphere at a heating rate of 10 K min^−1^. As shown in Figure 3, **NTPD** exhibits an onset decomposition temperature of 206 °C. Among the diazo compounds listed in Table 1, **NTPD** displays the highest onset decomposition temperature. Compared with the typical diazo compound DDNP, **NTPD** shows an increase of approximately 50 °C in onset decomposition temperature, indicating its enhanced thermal stability. This enhanced thermal stability can be attributed to its rigid molecular framework and stabilizing intra- and intermolecular hydrogen-bonding interactions. The DSC profile shows two exothermic peaks at 212 and 256 °C, and the TG curve exhibits the corresponding successive mass-loss steps, indicating that the decomposition of **NTPD** follows a distinct multistep pathway rather than a simple single-step process. The initial exothermic event at around 212 °C may be tentatively attributed to the preferential decomposition of the diazo functionality, whereas the more intense exothermic peak at 256 °C is attributed to the further decomposition of intermediate species formed in the primary stage.

Mechanical sensitivity is an important criterion for assessing the safety of energetic materials. The impact and friction sensitivities of **NTPD** were measured using standard BAM fall-hammer and friction methods, yielding values of 7 J and 168 N, respectively. Compared with other diazo compounds summarized in Table 1, **NTPD** exhibits relatively low mechanical sensitivity, with impact and friction sensitivities comparable to those of RDX (IS = 7.4 J, FS = 120 N) and significantly lower than those of DDNP (IS = 1 J, FS = 5 N). This favorable mechanical insensitivity can be attributed to the combined effects of intra- and intermolecular hydrogen-bonding interactions and the delocalized π-conjugated system, which enhance structural stability and mitigate localized responses to external mechanical stimuli. Collectively, **NTPD** exhibits a desirable combination of good thermal stability and low mechanical sensitivity, demonstrating its promising safety characteristics. 

### 2.4. Energetic Properties

Detonation performance, particularly detonation velocity and detonation pressure, is among the most important metrics for evaluating the energy output of energetic materials, and is primarily governed by density and enthalpy of formation [28,29]. As shown in Table 1, **NTPD** has a crystal density of 1.79 g cm^−3^, comparable to those of the other diazo compounds listed. Its enthalpy of formation was calculated to be 105.4 kJ mol^−1^ using the G4(MP2)-6x [30] method with Gaussian 16 [31] (Revision C.01), based on a geometry-optimized structure with no imaginary frequencies. This positive value is close to that of the classical diazo energetic compound DDNP. Based on these data, the detonation velocity and detonation pressure of **NTPD** were calculated using EXPLO5 [32] (version 6.02) to be 7790 m s^−1^ and 23.5 GPa, respectively. Notably, the detonation velocity of NTPD is higher than that of DDNP (7685 m s^−1^). This enhancement is primarily attributed to the slightly higher density of **NTPD** relative to DDNP. 

**Table 1:** Physicochemical and energetic properties of **NTPD** compared with other diazo-oxide compounds and RDX.

Compounds	*ρ* ^a^ [g∙cm^−3^]	∆*H*_f_ ^b^ [kJ∙mol^−1^]	*T*_d_ ^c^ [°C]	*D* ^d^ [m∙s^−1^]	*P* ^e^ [GPa]	IS ^f^ [J]	FS ^g^ [N]
**NTPD**	1.79	105.4	206	7790	23.5	7	168
DDNP [13]	1.73	142.4	157	7685	24.1	1	5
DNPO [7]	1.77	129.1	151	8055	26.4	4.5	28
HBNBD [12]	1.80	−42.0	170	7900	26.3	1	16
DNBD [12]	1.79	184.1	168	7978	26.9	1	40
BDNM [13]	1.83	819.7	199	8653	31.1	1	20–40
BDNP [13]	1.85	987.2	150	8943	34.0	1	20
OPPD [14]	1.80	1069.7	181	8679	31.0	5	60
RDX [33]	1.82	92.6	230	8997	35.1	7.4	120

^a^ Density, measured by gas pycnometer (25 °C). ^b^ Heats of formation. ^c^ Decomposition temperature (onset). ^d^ Detonation velocity (calculated with EXPLO5 v6.02). ^e^ Detonation pressure (calculated with EXPLO5 v6.02). ^f^ Impact sensitivity. ^g^ Friction sensitivity.

### 2.5. Theoretical Calculation

**Figure 4:**
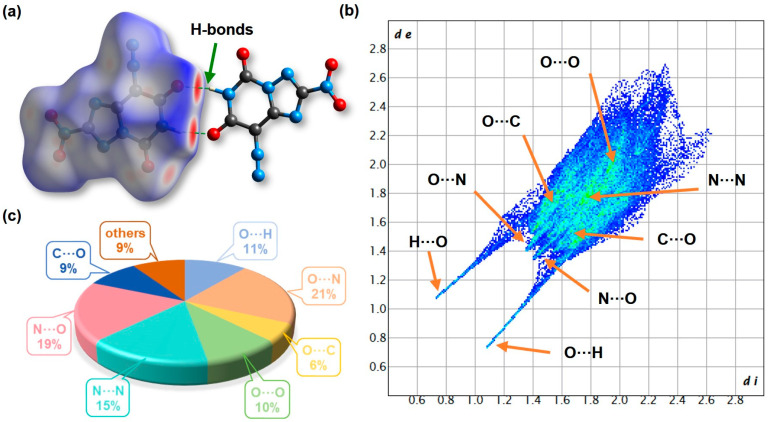
Hirshfeld surface analysis (**a**), 2D fingerprint plots (**b**), and percentage contributions of atomic-to-atomic contacts (**c**) for **NTPD**.

To gain deeper insights into the intermolecular interactions in **NTPD** and their potential influence on its molecular properties, Hirshfeld surface analysis, two-dimensional fingerprint plots, and the percentage contributions of intermolecular contacts were investigated using CrystalExplorer [34,35]. The Hirshfeld surface provides an intuitive visualization of intermolecular contacts in the crystal, where the blue, white, and red regions represent contacts longer than, close to, and shorter than the sum of the van der Waals radii, respectively. As shown in Figure 4a, the Hirshfeld surface of **NTPD** is relatively flat overall, which is consistent with the planarity of its molecular skeleton. The red regions are mainly distributed along the edges of the Hirshfeld surface, particularly around the O and H atoms involved in intermolecular hydrogen bonding (green dashed lines), indicating that these sites serve as key interaction hotspots in the crystal packing. The surface is dominated by blue and white regions, suggesting the presence of extensive van der Waals interactions in the crystal structure of **NTPD**. The two-dimensional fingerprint plots (Figure 4b) display characteristic sharp spikes corresponding to H···O/O···H interactions, whereas the dense central region is mainly associated with O···N, N···O, N···N, C···O, O···C, and O···O contacts, indicating that van der Waals interactions, in addition to hydrogen bonding, play a significant role in the crystal packing. Quantitative analysis (Figure 4c) further reveals that O···N (21%) and N···O (19%) make the largest contributions to the overall intermolecular interactions, which is consistent with the partially crossed arrangement observed in the single-crystal structure. These results demonstrate that the crystal packing of **NTPD** is collectively stabilized by hydrogen bonding and van der Waals interactions.

**Figure 5:**
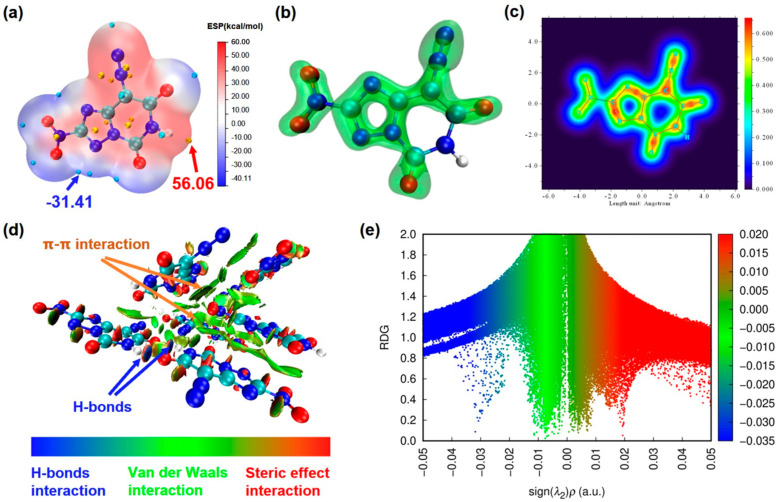
Molecular surface electrostatic potential (ESP) (**a**), isosurface plot of the π-electron localized orbital locator (LOL-π) (**b**), color-filled plane map of LOL-π (**c**), noncovalent interaction (NCI) analysis (**d**), and reduced density gradient (RDG) scatter plot (**e**) for **NTPD**.

To further elucidate the structure–property relationship of **NTPD**, molecular surface electrostatic potential (ESP) [36,37], π-electron localized orbital locator (LOL-π) [38], noncovalent interaction (NCI) and reduced density gradient (RDG) [39] analyses were performed using Multiwfn [40] 3.8 and VMD [41] v1.9.3 (Figure 5). As shown in Figure 5a, the ESP surface exhibits a pronounced electrostatic potential separation, with the most negative regions localized around the oxygen atoms (minimum ESP = −31.41 kcal mol^−1^) and the most positive regions distributed near the proton-bearing polar sites (maximum ESP = 56.06 kcal mol^−1^), indicating favorable donor–acceptor complementarity for intermolecular hydrogen-bond formation, consistent with the Hirshfeld surface and two-dimensional fingerprint analyses. Meanwhile, the LOL-π isosurface and the corresponding plane map (Figure 5b,c) reveal appreciable π-electron delocalization over the conjugated framework, suggesting that the planar molecular skeleton provides an appropriate electronic basis for intermolecular π–π interactions. These features are further supported by the NCI and RDG results. In the NCI isosurface map (Figure 5d), the blue regions correspond to relatively strong attractive interactions that can be assigned to intermolecular hydrogen bonds, whereas the broadly distributed green regions between adjacent molecules indicate extensive weak van der Waals interactions; notably, the green layered isosurfaces between neighboring aromatic frameworks also imply the presence of weak π–π contacts. Consistent with this, the RDG scatter plot (Figure 5e) shows blue spikes in the region of sign(λ_2_)ρ < 0, confirming attractive interactions such as hydrogen bonding, while the dense green scatter points around sign(λ_2_)ρ ≈ 0 reflect the substantial contribution of van der Waals interactions to the crystal packing. Collectively, the crystal packing of **NTPD** is stabilized by the synergistic interplay of hydrogen bonding, van der Waals interactions, and weak π–π contacts. Such a cooperative packing mode enhances the structural rigidity and packing stability of the crystal, thereby contributing to its excellent thermal stability and satisfactory sensitivity.

## 3. Materials and Methods 

Caution! The nitrogen-rich compounds described herein are energetic materials and may explode under certain conditions. Therefore, all operations, including preparation, handling, characterization, and performance testing, were conducted on a small scale by properly trained and grounded personnel in a protected fume hood behind a safety shield. Face shields and leather gloves were worn throughout. All reagents were of analytical grade and were used without further purification.

### 3.1. Reagents and Instruments 

**Reagents**: Cyanogen bromide (BrCN) and sodium nitrite were purchased from Shanghai Aladdin Biochemical Technology Co., Ltd. (Shanghai, China). Ethanol, methanol, sulfuric acid, and 80% hydrazine hydrate were purchased from Chengdu Kelong Chemical Co., Ltd. (Chengdu, China).

**Instruments**: ^1^H and ^13^C NMR spectra were recorded on a Bruker AVANCE 400 spectrometer (Bruker BioSpin, Ettlingen, Germany) at 400 and 100 MHz, respectively, using DMSO-d_6_ as the solvent and the residual solvent signals as internal references (^1^ H NMR: δ 2.50 ppm; ^13^C NMR: δ 39.52 ppm). Mass spectra were recorded on a Thermo QE Plus instrument (Thermo Fisher Scientific, Waltham, MA, USA) using APCI. Infrared (IR) spectra were collected on a Spectrum II spectrometer (PerkinElmer, Waltham, MA, USA) using KBr pellets. Elemental analyses (C, H, O, and N) were performed on a Vario EL III CHNOS device (Elementar Analysensysteme, Langenselbold, Germany). Single-crystal X-ray diffraction data were collected at room temperature on an Xcalibur diffractometer equipped with a CCD detector (Oxford Diffraction, Abingdon, UK) using Cu-Ka radiation (λ = 1.54178 Å) with ω scans. Thermal analysis was carried out on a TG-DSC analyzer equipped with an auto-cooling accessory (Mettler-Toledo, Greifensee, Switzerland). Impact and friction sensitivities were measured using a standard BAM fall hammer and a BAM friction tester, respectively (OZM, Prague, Czech Republic).

### 3.2. Synthesis of 4-Chloro-6-hydrazineylpyrimidine-2,5-diamine (CHPD)

4,6-Dichloropyrimidine-2,5-diamine (1.79 g, 10 mmol) was suspended in methanol (30 mL) and refluxed at 80 °C for 10 min, during which the solid did not dissolve completely. Furthermore, 80% hydrazine hydrate (0.61 mL, 10 mmol) was then added, and the reaction mixture was further refluxed at 80 °C for about 1 h. During this period, a large amount of pink solid gradually precipitated. After the mixture had cooled to room temperature, the precipitate was collected by filtration, washed with ethanol, and dried to afford 4-chloro-6-hydrazineylpyrimidine-2,5-diamine (CHPD) as a pink solid in 73% yield. ^1^H NMR (400 MHz, d_6_-DMSO): δ 7.78(1H), 5.71(2H), 4.29(2H), 3.93(2H) ppm. ^13^C NMR (100 MHz, d_6_-DMSO): δ 156.0, 155.4, 139.8, 112.6 ppm. MS (APCI^−^): 173.03:175.03 = 3:1 [C_4_H_7_ClN_6_ − H]^−^. IR (KBr, cm^−1^): 3294, 3151, 1635, 1566, 1445, 1257, 1150, 1011, 856. Elemental analysis calcd (%) for C_4_H_7_ClN_6_: C 27.5, H 4.0, N 48.1. Found: C 27.3, H 4.3, N 48.0.

### 3.3. Synthesis of 7-Chloro-[1,2,4]triazolo[4,3-c]pyrimidine-3,5,8-triamine (CTPT)

CHPD (1.75 g, 10 mmol) was suspended in methanol (50 mL) and heated under reflux at 80 °C for 10 min, during which complete dissolution was not achieved. Cyanogen bromide (1.06 g, 10 mmol) was dissolved in methanol (2 mL) and added dropwise to the suspension, and the resulting mixture was further refluxed at 80 °C for about 1 h. During the reaction, a deep purple solid gradually precipitated. After cooling to room temperature, the precipitate was collected by filtration, washed with water and ethanol, and dried to afford 7-chloro-[1,2,4]triazolo[4,3-c]pyrimidine-3,5,8-triamine (CTPT) as a deep purple solid in 54% yield. ^1^H NMR (400 MHz, d_6_-DMSO): δ 6.82(1H), 6.04(1H), 4.70(1H) ppm. ^13^C NMR (100 MHz, d_6_-DMSO): δ 148.0, 144.5, 137.6, 119.5, 117.8 ppm. MS (APCI^−^): 198.03:200.03 = 3:1 [C_5_H_6_ClN_7_ − H]^−^. IR (KBr, cm^−1^): 3059, 1686, 1607, 1576, 1543, 1457, 1355, 1271, 1034, 1007, 854. Elemental analysis calcd (%) for C_5_H_6_ClN_7_: C 30.1, H 3.0, N 49.1. Found: C 29.9, H 3.2, N 48.8.

### 3.4. Synthesis of 2-Nitro-5-oxo[1,2,4]triazolo[1,5-c]pyrimidin-8-diazonium-7-olate (NTPD)

CTPT (0.20 g, 1 mmol) was suspended in 20 wt% aqueous sulfuric acid (5 mL) and maintained at 0 °C for 10 min. Sodium nitrite (9.66 g, 140 mmol) was dissolved in water (15 mL) and likewise kept at 0 °C for 10 min. The sulfuric acid suspension of compound 2 was then added dropwise to the aqueous sodium nitrite solution, and the resulting mixture was stirred at 0 °C for 20 min after the addition was complete. The reaction mixture was subsequently allowed to warm to room temperature and stirred for a further 5 h, after which it was acidified to pH ~3 with 20 wt% aqueous sulfuric acid. The mixture was extracted with ethyl acetate (3–4 times), and the combined organic layers were washed with saturated aqueous NaCl solution (2–3 times), dried over anhydrous calcium carbonate, and filtered. Removal of the solvent under reduced pressure afforded 2-nitro-5-oxo[1,2,4]triazolo[1,5-c]pyrimidin-8-diazonium-7-olate (**NTPD**) as a pale yellow solid in 35% yield. ^1^H NMR (400 MHz, d_6_-DMSO): δ 12.87 ppm. ^13^C NMR (100 MHz, d_6_-DMSO): δ 162.7, 159.1, 149.7, 142.7, 66.1 ppm. IR (KBr, cm^−1^): 3147, 3028, 2846, 2198, 2165, 1754, 1664, 1564, 1492, 1396, 1294, 1202, 835, 731, 623. Elemental analysis calcd (%) for C_5_HN_7_O_4_: C 26.9, H 0.5, N 44.0, O 28.7. Found: C 26.5, H 0.7, N 43.4, O 29.4.

## 4. Conclusions

In summary, a new fused diazonium inner salt, 2-nitro-5-oxo[1,2,4]triazolo[1,5-c]pyrimidin-8-diazonium-7-olate (**NTPD**), was synthesized through a concise three-step route. Single-crystal X-ray diffraction revealed that the formation of **NTPD** involved a Dimroth rearrangement, yielding a fused [1,2,4]triazolo[1,5-c]pyrimidine framework. **NTPD** exhibits a desirable combination of high thermal stability and reduced mechanical sensitivity, exhibiting an onset decomposition temperature of 206 °C, as well as impact and friction sensitivities of 7 J and 168 N, respectively, while maintaining competitive detonation performance. Structural and theoretical analyses further indicate that the nearly planar fused skeleton, together with the synergistic effects of hydrogen bonding, van der Waals interactions, and weak π–π contacts, enhances molecular rigidity and crystal packing stability, thereby accounting for its favorable safety profile. These findings provide useful insight into the design of fused diazonium inner salts with improved thermal stability and reduced sensitivity.

## Data Availability

CCDC 2547065 (**NTPD**) contains the supplementary crystallographic data for this paper. These data can be obtained free of charge via www.ccdc.cam.ac.uk/data_request/cif (accessed on 17 April 2026) or by emailing data_request@ccdc.cam.ac.uk.

## Supplementary Materials

The following supporting information can be downloaded at: https://www.mdpi.com/article/10.3390/molecules31132340/s1, Figure S1: The asymmetric unit of **NTPD**; Figure S2: ^1^H NMR spectrum of 4-chloro-6-hydrazineylpyrimidine-2,5-diamine (CHPD) in DMSO-*d*6 at 400 MHz; Figure S3: ^13^C NMR spectrum of 4-chloro-6-hydrazineylpyrimidine-2,5-diamine (CHPD) in DMSO-*d*6 at 100 MHz; Figure S4: ^1^H NMR spectrum of 7-chloro-[1,2,4]triazolo[4,3-c]pyrimidine-3,5,8-triamine (CTPT) in DMSO-*d*6 at 400 MHz; Figure S5: ^13^C NMR spectrum of 7-chloro-[1,2,4]triazolo[4,3-c]pyrimidine-3,5,8-triamine (CTPT) in DMSO-*d*6 at 100 MHz; Figure S6: ^1^H NMR spectrum of 2-nitro-5-oxo[1,2,4]triazolo[1,5-c]pyrimidin-8-diazonium-7-olate (**NTPD**) in DMSO-*d*6 at 400 MHz; Figure S7: ^13^C NMR spectrum of 2-nitro-5-oxo[1,2,4]triazolo[1,5-c]pyrimidin-8-diazonium-7-olate (**NTPD**) in DMSO-*d*6 at 100 MHz; Figure S8: IR spectrum of 4-chloro-6-hydrazineylpyrimidine-2,5-diamine (CHPD); Figure S9: IR spectrum of 7-chloro-[1,2,4]triazolo[4,3-c]pyrimidine-3,5,8-triamine (CTPT); Figure S10: IR spectrum of 2-nitro-5-oxo[1,2,4]triazolo[1,5-c]pyrimidin-8-diazonium-7-olate (**NTPD**); Figure S11: Mass spectrum of 4-chloro-6-hydrazineylpyrimidine-2,5-diamine (CHPD). Figure S12: Mass spectrum of 7-chloro-[1,2,4]triazolo[4,3-c]pyrimidine-3,5,8-triamine (CTPT). Figure S13: Proposed reaction mechanism of converting CTPT to NTPD. Table S1: Crystal data and structure refinement for **NTPD**; Table S2: Bond Lengths for **NTPD**; Table S3: Bond Angles for **NTPD**; Table S4: Torsion Angles for **NTPD**. Refs. [42,43,44,45,46,47,48] are cited in the Supplementary Materials.
