# Supplementary material for: Synthesis, Structure and Performance of an Insensitive Diazonium Inner Salt Energetic Material
Source: Molecules. 2026 Jul 3;31(13):2340. doi: 10.3390/molecules31132340 (PMC13362582; doi:10.3390/molecules31132340)
Supplement: Supplementary file 1 [file molecules-31-02340-s001.zip › molecules-4372866-supplementary.pdf]

Supporting Information

Synthesis, Structure and Performance of an Insensitive Diazonium Inner Salt Energetic Material

Haifeng Wang [1], Jinxin Wang [1,2], Ruibing Lv [1], Yapeng Yao [1], Pengzhao Han [1], Wenquan Zhang [1] and Kangcai Wang [1,*]

[1] National Key Laboratory of Chemical Explosion Safety, Institute of Chemical Materials, China Academy of Engineering Physics (CAEP), Mianyang 621900, China; wanghaifeng24@gscaep.ac.cn (H.W.); wangjinxin@casc42.cn (J.W.); lvruibing0719@163.com (R.L.); yaoyp21@163.com (Y.Y.); hpzsdbrk12138@163.com (P.H.); zhangwq-cn@caep.cn (W.Z.)

[2] National Key Laboratory of Aerospace Chemical Power, Xiangyang 441003, China

* Correspondence: wangkangcai@caep.cn

Table of Contents

Section S1. Crystallographic detail 2
Section S2. Theoretical calculation method of heats of formation 6
Section S3. ^{1}H and ^{13}C NMR spectra for all compounds 6
Section S4. IR spectra of all compounds 9
Section S5. Mass spectra for CHPD and CTPT 11
Section S6. Proposed reaction mechanism of converting CTPT to NTPD 12
References 12

Section S1. Crystallographic detail

Table S1 Crystal data and structure refinement for **NTPD**.

Crystal	**NTPD**
CCDC	2547065
Empirical formula	$C_5HN_7O_4$
Formula weight	223.13
Temperature/K	292.29(10)
Crystal system	triclinic
Space group	*P*-1
a/Å	8.04740(10)
b/Å	9.67010(10)
c/Å	11.36110(10)
α/°	74.0100(10)
β/°	87.9250(10)
γ/°	77.2730(10)
Volume/Å^3	828.741(16)
Z	4
ρ_{calc}g/cm^3	1.788
μ/mm^{-1}	1.389
F(000)	448.0
Crystal size/mm^3	0.18 × 0.18 × 0.16
Radiation	Cu Kα (λ = 1.54184)
2Θ range for data collection/°	8.098 to 155.326
Index ranges	-10 ≤ h ≤ 10, -12 ≤ k ≤ 12, -14 ≤ l ≤ 14
Reflections collected	26400
Independent reflections	3479 [R_{int} = 0.0417, R_{sigma} = 0.0186]
Data/restraints/parameters	3479/6/297
Goodness-of-fit on F^2	1.091
Final R indexes [I>=2σ (I)]	R_1 = 0.0386, wR_2 = 0.1026
Final R indexes [all data]	R_1 = 0.0547, wR_2 = 0.1235
Largest diff. peak/hole / e Å^{-3}	0.40/-0.35

Table S2 Bond Lengths for **NTPD**.

Atom	Atom	Length/Å	Atom	Atom	Length/Å
O8	C10	1.196(2)	N12	C10	1.373(2)
O4	C2	1.223(2)	N12	C9	1.385(2)
O5	N8	1.2237(19)	N3	N2	1.371(2)
O6	N8	1.2178(19)	N3	C4	1.368(2)
O3	C1	1.202(2)	N3	C1	1.402(2)
N11	N10	1.3679(17)	O2	N1	1.212(2)
N11	C7	1.362(2)	N2	C5	1.306(2)
N11	C10	1.415(2)	O1	N1	1.215(2)
O7	C9	1.216(2)	N5	C2	1.391(2)
N10	C6	1.306(2)	N5	C1	1.369(3)
N9	C7	1.319(2)	N1	C5	1.457(2)
N9	C6	1.352(2)	N13	C8	1.320(2)
N6	N7	1.105(2)	N13	N14	1.103(2)
N6	C3	1.343(2)	C7	C8	1.418(2)
N4	C4	1.318(2)	C3	C4	1.419(2)
N4	C5	1.352(2)	C3	C2	1.441(2)
N8	C6	1.4602(19)	C8	C9	1.433(2)

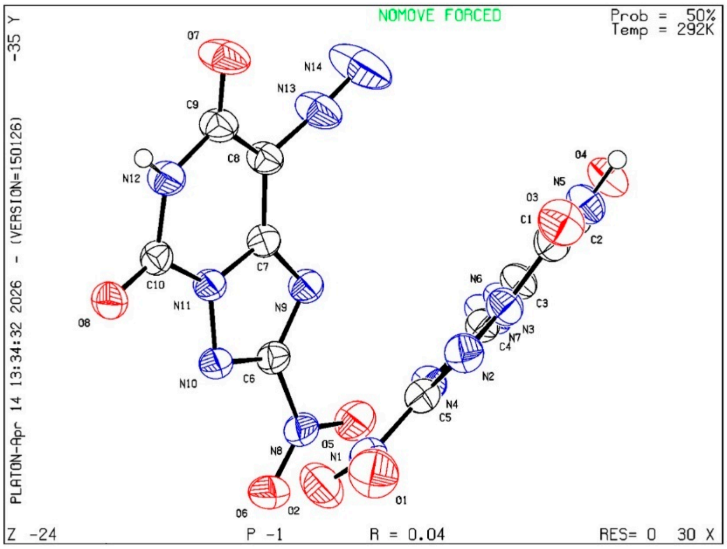

Figure S1 The asymmetric unit of **NTPD**.

Table S3 Bond Angles for **NTPD**.

Atom	Atom	Atom	Angle/°	Atom	Atom	Atom	Angle/°
N10	N11	C10	122.90(13)	N9	C6	N8	120.99(14)
C7	N11	N10	110.01(12)	N6	C3	C4	119.73(14)
C7	N11	C10	127.02(13)	N6	C3	C2	118.89(16)
C6	N10	N11	99.47(12)	C4	C3	C2	121.38(16)
C7	N9	C6	100.17(13)	O8	C10	N11	122.82(14)
N7	N6	C3	179.56(17)	O8	C10	N12	125.42(15)
C4	N4	C5	100.76(14)	N12	C10	N11	111.76(13)
O5	N8	C6	116.51(14)	N4	C4	N3	110.23(15)
O6	N8	O5	125.51(14)	N4	C4	C3	132.88(16)
O6	N8	C6	117.98(14)	N3	C4	C3	116.89(15)
C10	N12	C9	128.54(14)	N13	C8	C7	121.69(15)
N2	N3	C1	123.18(15)	N13	C8	C9	115.98(15)
C4	N3	N2	110.11(14)	C7	C8	C9	121.27(14)
C4	N3	C1	126.07(15)	O4	C2	N5	120.48(15)
C5	N2	N3	99.52(14)	O4	C2	C3	125.73(16)
C1	N5	C2	128.91(15)	N5	C2	C3	113.77(16)
O2	N1	O1	125.46(18)	N4	C5	N1	120.99(16)
O2	N1	C5	116.95(16)	N2	C5	N4	119.38(16)
O1	N1	C5	117.59(18)	N2	C5	N1	119.63(17)
N14	N13	C8	175.7(2)	O3	C1	N3	122.92(18)
N11	C7	C8	116.33(13)	O3	C1	N5	124.66(17)
N9	C7	N11	110.72(13)	N5	C1	N3	112.42(15)
N9	C7	C8	132.94(15)	O7	C9	N12	121.75(16)
N10	C6	N9	119.63(13)	O7	C9	C8	123.65(16)
N10	C6	N8	119.38(14)	N12	C9	C8	114.60(14)

Table S4 Torsion Angles for **NTPD**.

A	B	C	D	Angle/°	A	B	C	D	Angle/°
O5	N8	C6	N10	179.53(15)	C7	N11	C10	N12	-1.9(2)
O5	N8	C6	N9	-0.1(2)	C7	N9	C6	N10	0.23(19)
O6	N8	C6	N10	0.4(2)	C7	N9	C6	N8	179.83(14)
O6	N8	C6	N9	-179.15(15)	C7	C8	C9	O7	171.75(18)
N11	N10	C6	N9	-0.20(19)	C7	C8	C9	N12	-7.9(3)
N11	N10	C6	N8	-179.81(14)	C6	N9	C7	N11	-0.15(17)
N11	C7	C8	N13	175.10(16)	C6	N9	C7	C8	178.77(18)
N11	C7	C8	C9	7.3(2)	C10	N11	N10	C6	-176.98(14)
N10	N11	C7	N9	0.05(18)	C10	N11	C7	N9	176.96(15)
N10	N11	C7	C8	-179.07(14)	C10	N11	C7	C8	-2.2(2)
N10	N11	C10	O8	-5.2(2)	C10	N12	C9	O7	-176.05(19)
N10	N11	C10	N12	174.61(14)	C10	N12	C9	C8	3.6(3)
N9	C7	C8	N13	-3.8(3)	C4	N4	C5	N2	0.8(2)
N9	C7	C8	C9	-171.54(17)	C4	N4	C5	N1	-179.73(14)
N6	C3	C4	N4	-3.7(3)	C4	N3	N2	C5	0.70(18)
N6	C3	C4	N3	177.22(15)	C4	N3	C1	O3	170.07(18)
N6	C3	C2	O4	0.2(3)	C4	N3	C1	N5	-9.2(2)
N6	C3	C2	N5	179.25(15)	C4	C3	C2	O4	179.44(17)
N3	N2	C5	N4	-1.0(2)	C4	C3	C2	N5	-1.5(2)
N3	N2	C5	N1	179.56(14)	C2	N5	C1	O3	-174.15(18)
O2	N1	C5	N4	-11.3(2)	C2	N5	C1	N3	5.1(3)
O2	N1	C5	N2	168.15(17)	C2	C3	C4	N4	177.04(17)
N2	N3	C4	N4	-0.31(19)	C2	C3	C4	N3	-2.0(2)
N2	N3	C4	C3	178.97(14)	C5	N4	C4	N3	-0.23(17)
N2	N3	C1	O3	0.2(3)	C5	N4	C4	C3	-179.35(18)
N2	N3	C1	N5	-179.10(15)	C1	N3	N2	C5	172.00(16)
O1	N1	C5	N4	169.20(17)	C1	N3	C4	N4	-171.30(16)
O1	N1	C5	N2	-11.3(2)	C1	N3	C4	C3	8.0(3)
N13	C8	C9	O7	3.3(3)	C1	N5	C2	O4	178.85(18)
N13	C8	C9	N12	-176.35(17)	C1	N5	C2	C3	-0.3(3)
C7	N11	N10	C6	0.08(16)	C9	N12	C10	O8	-179.13(17)
C7	N11	C10	O8	178.28(15)	C9	N12	C10	N11	1.1(3)

Section S2. Theoretical calculation method of heats of formation

The enthalpy (ΔH) for 2-nitro-5-oxo[1,2,4]triazolo[1,5-c]pyrimidin-8-diazonium-7-olate (**NTPD**) was directly calculated by G4(MP2)-6x[1] method with Gaussian 16[2] (Revision C.01) suite of program. In this method, geometries were optimized with the BMK[3] functional using the 6-31+G(2df,p) basis set. Zero-point vibrational energies (ZPVEs) and thermal corrections to ΔH at 298K, derived from scaled BMK/6-31+G(2df,p)[4] frequencies, were incorporated into the total energies. Single-point energies were obtained at the HF/GFHFB3, HF/GFHFB4, MP2(FrzG4)/GTMP2LargeXP, and CCSD (T,FrzG4)/GTBas1 levels with composite procedures.

After acquiring the enthalpy of compounds, their gas phase enthalpy of formation can be calculated by atomization method as following equation (1):

$$\Delta H_f(g) = H\big(C_l H_m O_n N_i(g)\big) - lH\big(C(g)\big) - \frac{m}{2}H\big(H_2(g)\big) - \frac{n}{2}H\big(O_2(g)\big)$$

$$-\frac{i}{2}H\big(N_2(g)\big) + lH_{vap}(graphite) \qquad \textbf{(Equation S1)}$$

For neutral compounds with calculated gas state heat of formation, the solid-state heat of formation was calculated by the following equation 2. The heat of sublimation ΔH_{SUB} was estimated by the following equation 3. T is the decomposition point temperature in Kelvin.

$$\Delta H_f(\mathrm{s}) = \Delta H_f(\mathrm{g}) - \Delta H_{SUB} \qquad \textbf{(Equation S2)}$$

$$\Delta H_{SUB} = 0.188 * T \qquad \textbf{(Equation S3)}$$

Section S3. ^{1}H and ^{13}C NMR spectra for all compounds

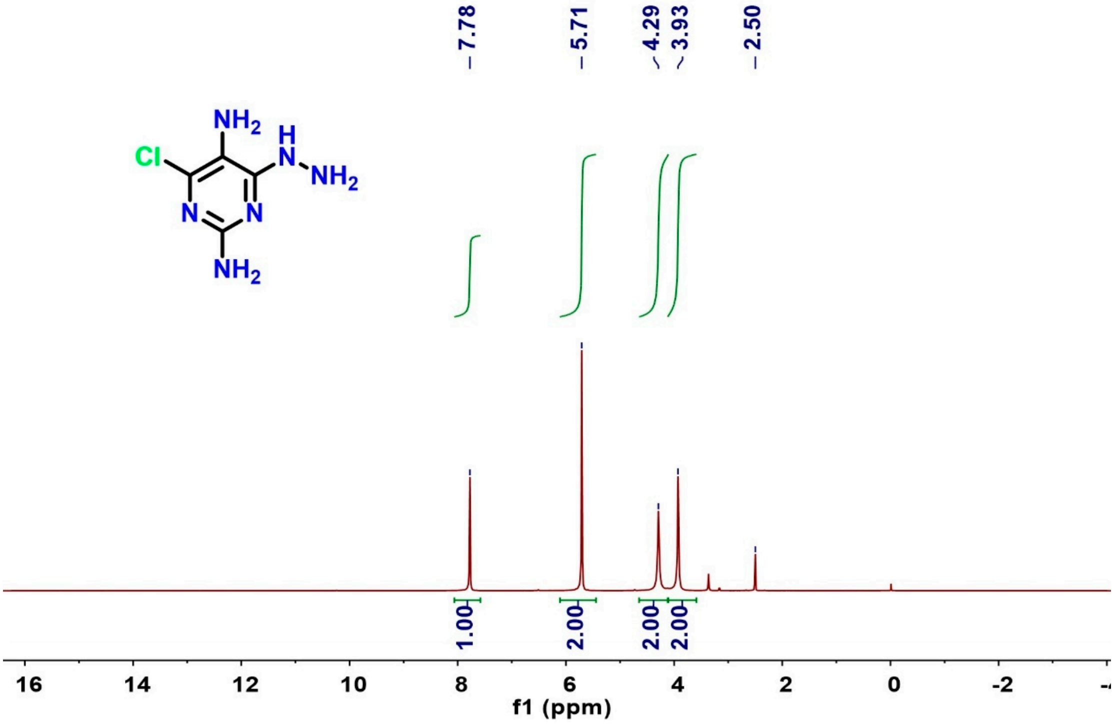

Figure S2 ^{1}H NMR spectrum of 4-chloro-6-hydrazineylpyrimidine-2,5-diamine (CHPD) in DMSO-*d6* at 400 MHz.

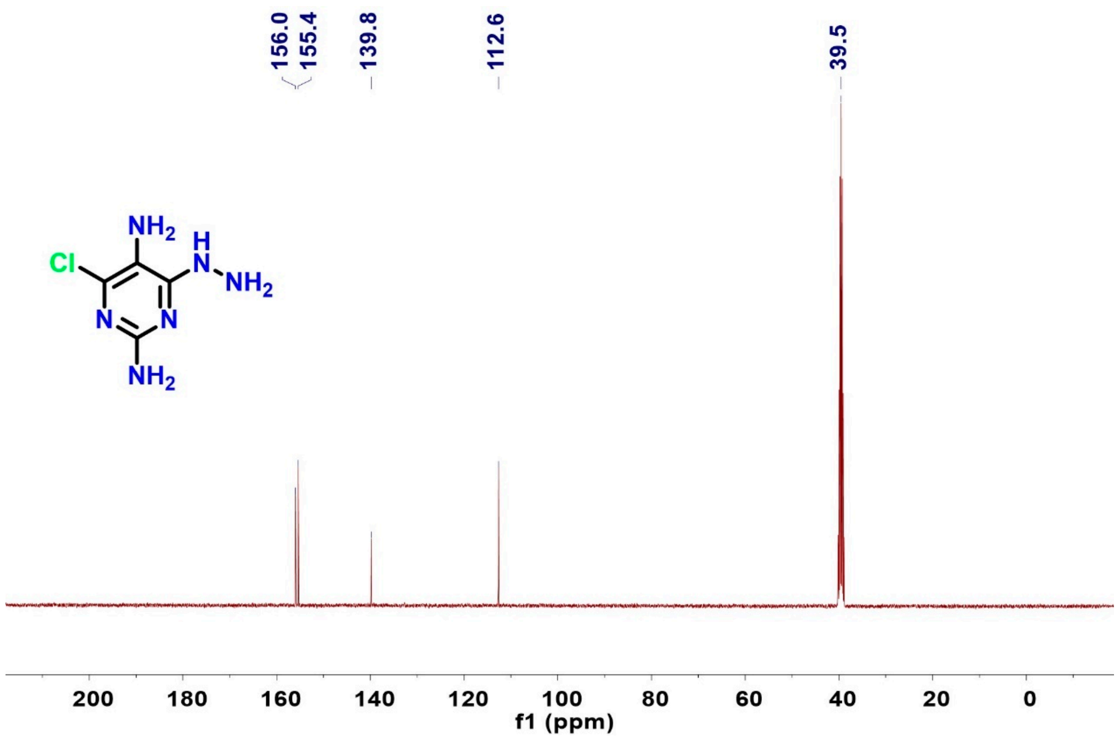

Figure S3 ^{13}C NMR spectrum of 4-chloro-6-hydrazineylpyrimidine-2,5-diamine (CHPD) in DMSO-*d6* at 100 MHz.

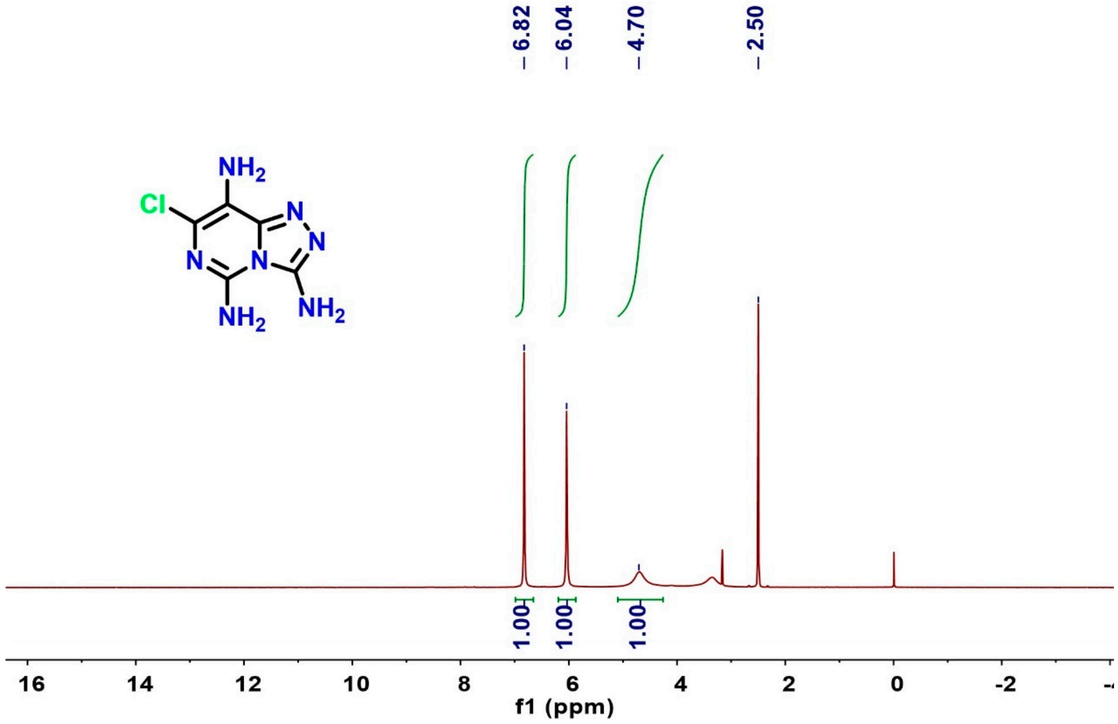

Figure S4 ^{1}H NMR spectrum of 7-chloro-[1,2,4]triazolo[4,3-c]pyrimidine-3,5,8-triamine (CTPT) in DMSO-*d6* at 400 MHz.

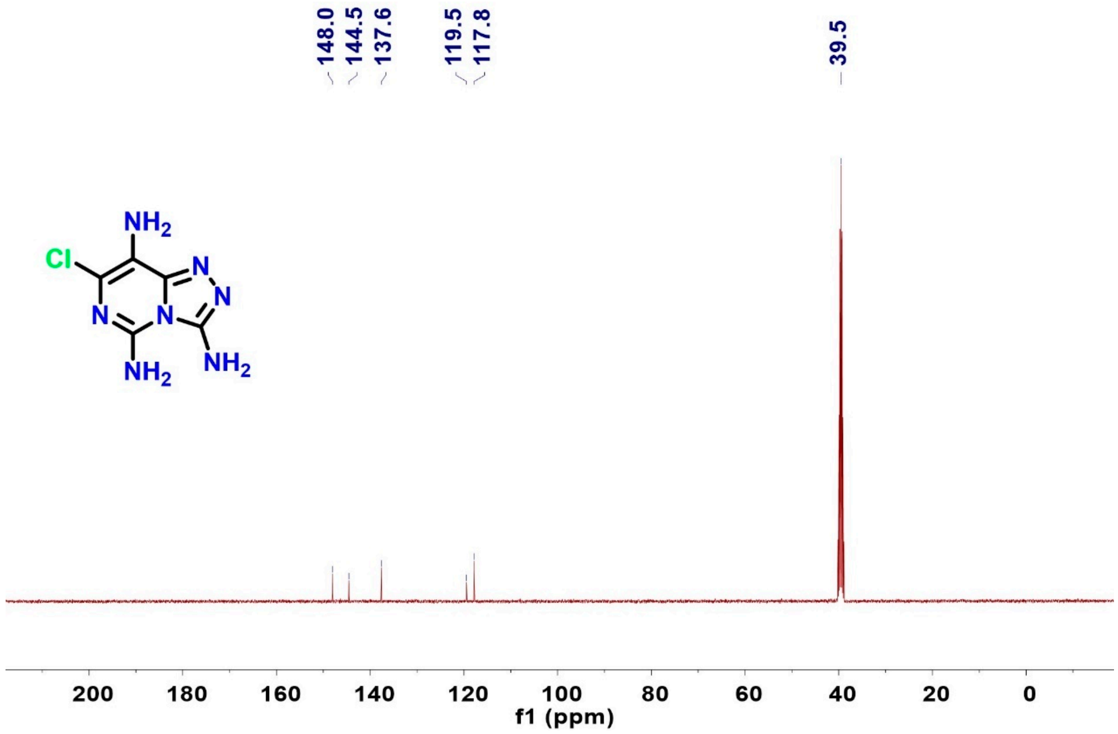

Figure S5 ^{13}C NMR spectrum of 7-chloro-[1,2,4]triazolo[4,3-c]pyrimidine-3,5,8-triamine (CTPT) in DMSO-*d*6 at 100 MHz.

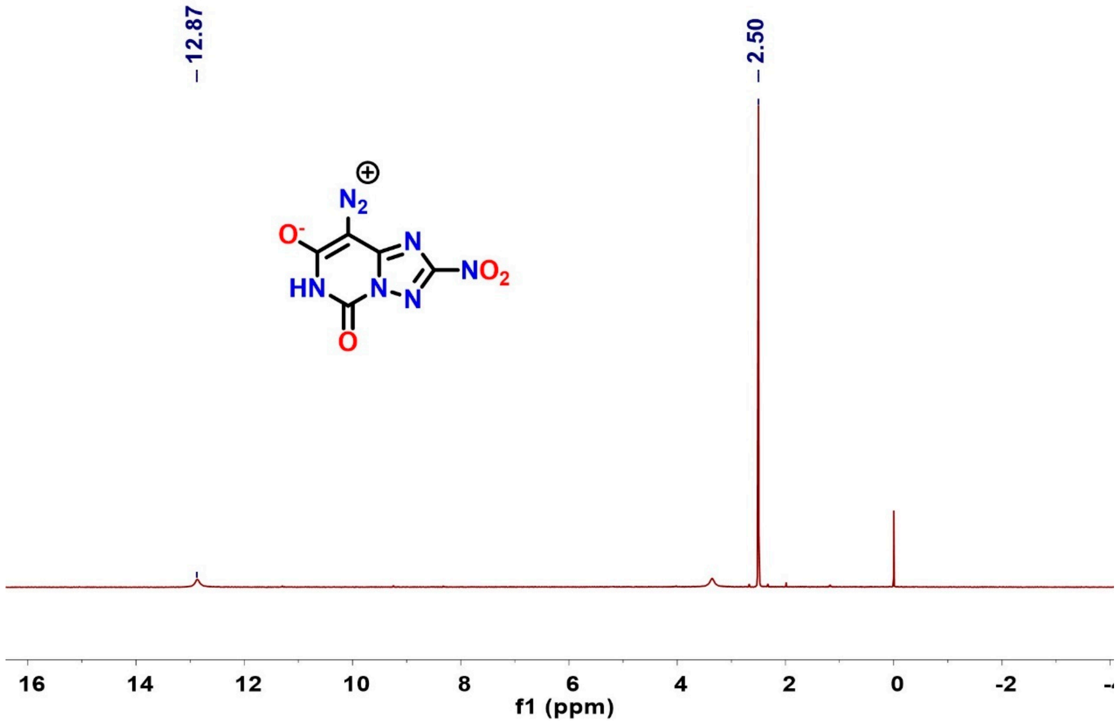

Figure S6 ^{1}H NMR spectrum of 2-nitro-5-oxo[1,2,4]triazolo[1,5-c]pyrimidin-8-diazonium-7-olate (**NTPD**) in DMSO-*d*6 at 400 MHz.

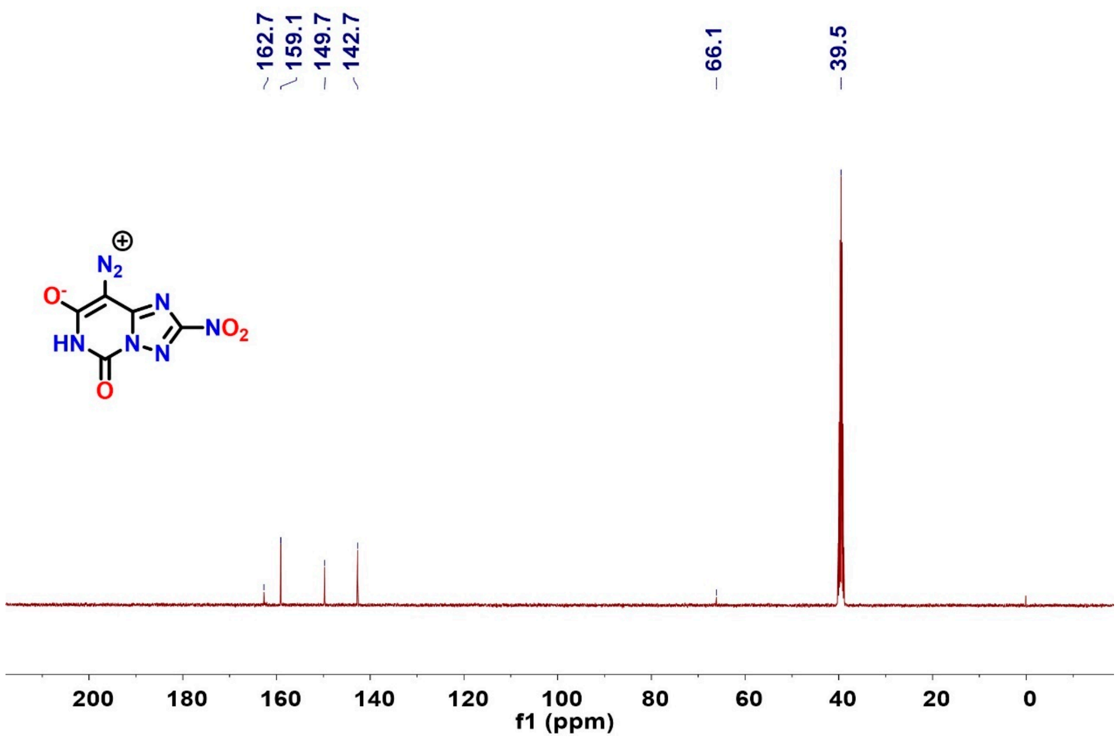

Figure S7 ^{13}C NMR spectrum of 2-nitro-5-oxo[1,2,4]triazolo[1,5-c]pyrimidin-8-diazonium-7-olate (**NTPD**) in DMSO-*d6* at 100 MHz.

Section S4. IR spectra of all compounds

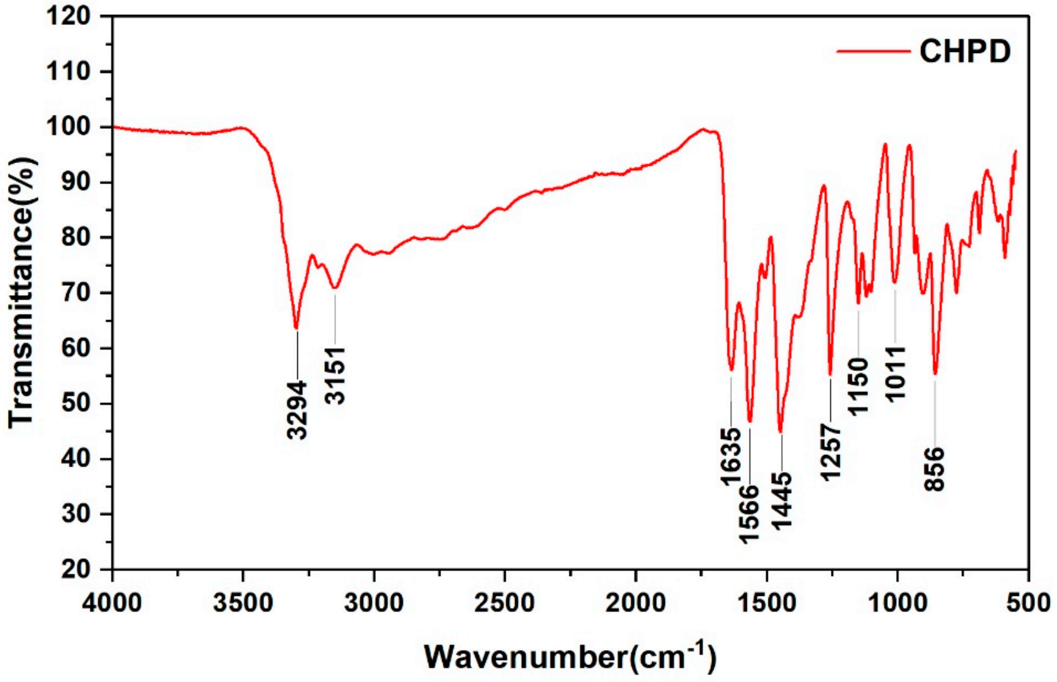

Figure S8 IR spectrum of 4-chloro-6-hydrazineylpyrimidine-2,5-diamine (CHPD).

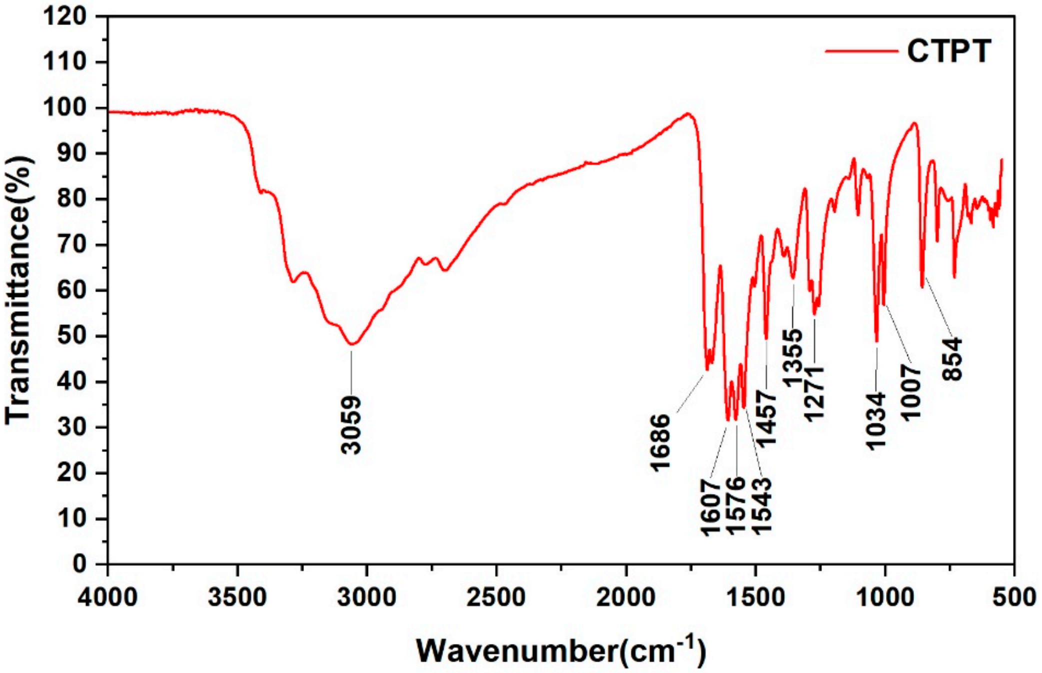

Figure S9 IR spectrum of 7-chloro-[1,2,4]triazolo[4,3-c]pyrimidine-3,5,8-triamine (CTPT).

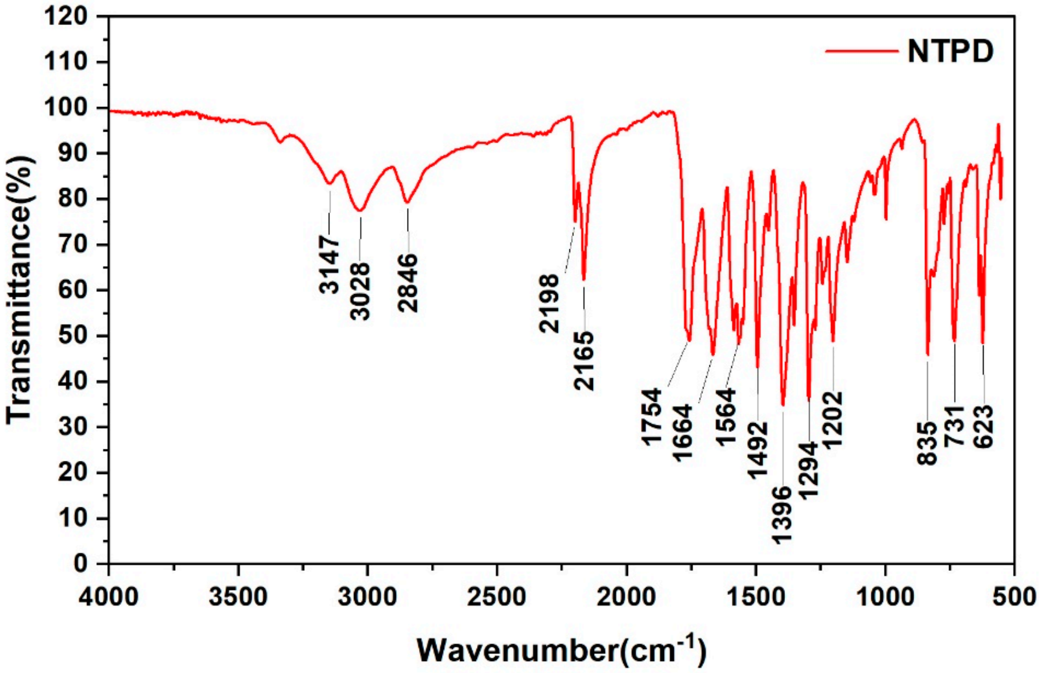

Figure S10 IR spectrum of 2-nitro-5-oxo[1,2,4]triazolo[1,5-c]pyrimidin-8-diazonium-7-olate (**NTPD**).

Section S5. **Mass spectra for CHPD and CTPT**

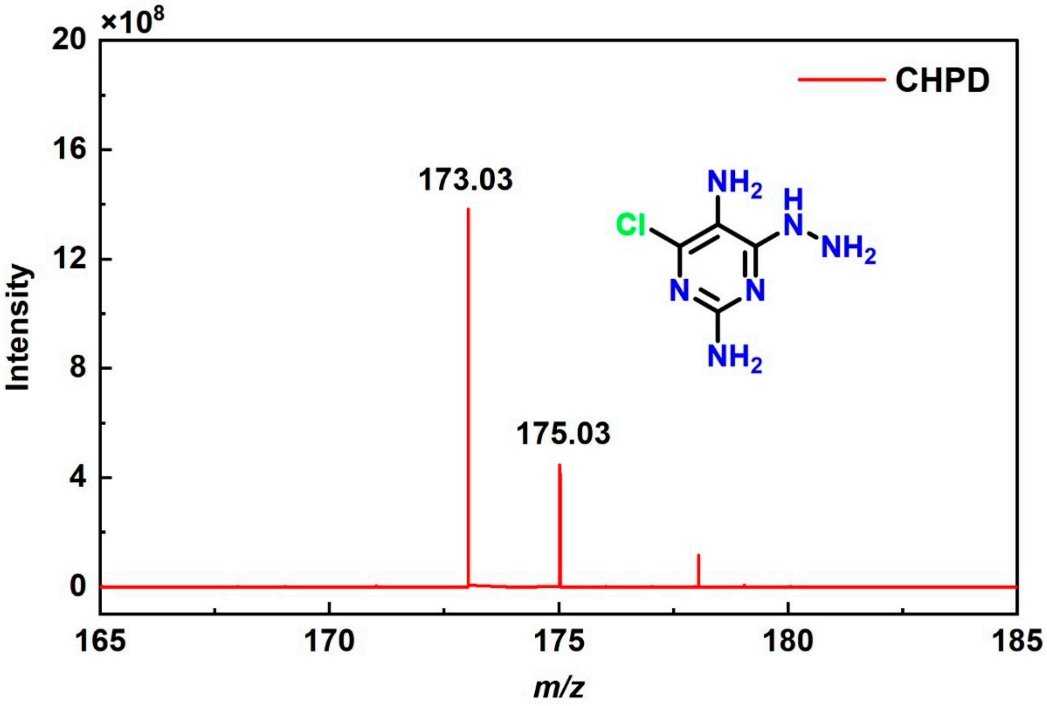

Figure S11 Mass spectrum of 4-chloro-6-hydrazineylpyrimidine-2,5-diamine (CHPD).

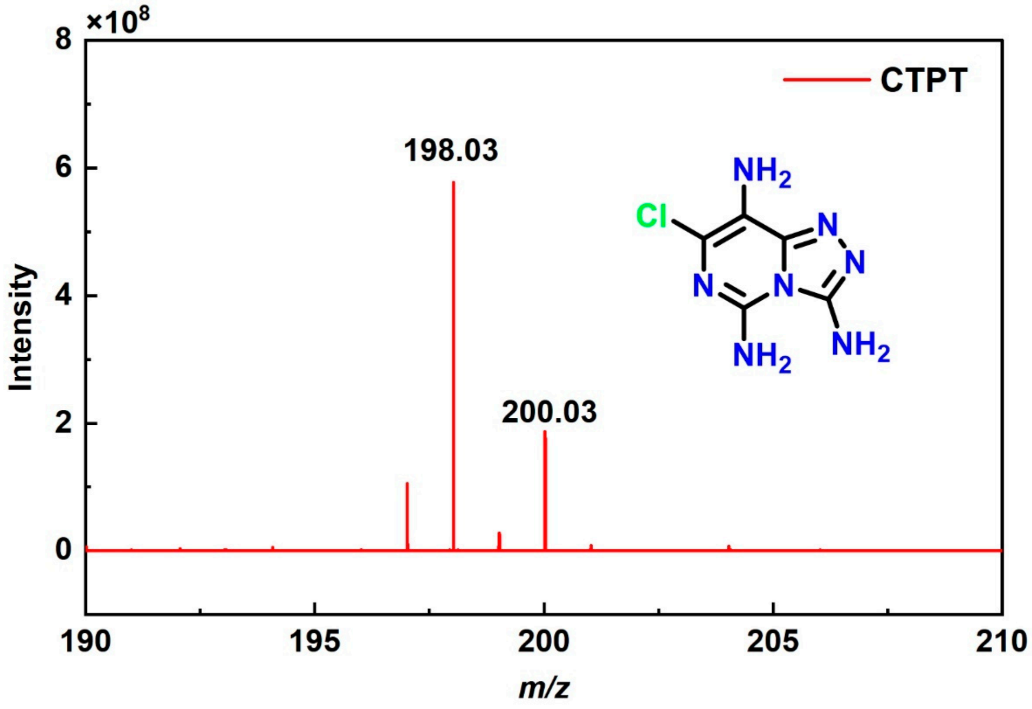

Figure S12 Mass spectrum of 7-chloro-[1,2,4]triazolo[4,3-c]pyrimidine-3,5,8-triamine (CTPT).

Section S6. Proposed reaction mechanism of converting CTPT to NTPD

The reaction is proposed to begin with acid-promoted hydrolysis of the chloro substituent on the electron-deficient heteroaromatic ring to give a hydroxy-substituted intermediate [5]. Under acidic $NaNO_2$ conditions, the exocyclic amino groups may be converted into diazonium-type intermediates through nitrosation/diazotization [6]. The drawn multi-diazotized structure is intended only to indicate possible reactive sites rather than a confirmed discrete intermediate. Subsequently, one diazonium-type moiety may be stabilized as a zwitterionic inner-salt form with the adjacent hydroxy/oxo-anionic site [7]; another may undergo hydrolytic dediazoniation followed by hydroxy–oxo tautomerization to afford the carbonyl group [8,9]; and a third may undergo nitrite-mediated dediazoniative nitration to give the nitro-substituted product [10]. Finally, the initially formed fused triazolopyrimidine isomer may undergo Dimroth rearrangement to afford the thermodynamically more stable NTPD [11].

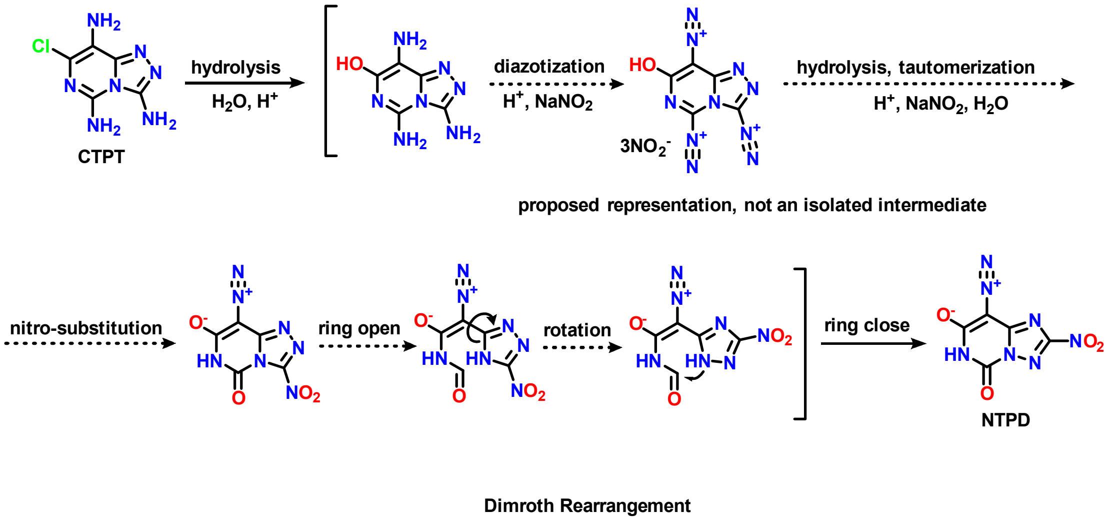

Figure S13 Proposed reaction mechanism of converting CTPT to NTPD.

References

1. Chan, B.; Karton, A.; Raghavachari, K. G4(MP2)-XK: A Variant of the G4(MP2)-6X Composite Method with Expanded Applicability for Main-Group Elements up to Radon. *J. Chem. Theory Comput.* **2019,** *15*. https://doi.org/10.1021/acs.jctc.9b00449

2. Frisch, M. J.; Trucks, G. W.; Schlegel, H. B.; Scuseria, G. E.; Robb, M. A.; Cheeseman, J. R.; Scalmani, G.; Barone, V.; Petersson, G. A.; Nakatsuji, H.; Li, X.; Caricato, M.; Marenich, A. V.; Bloino, J.; Janesko, B. G.; Gomperts, R.; Mennucci, B.; Hratchian, H. P.; Ortiz, J. V.; Izmaylov, A. F.; Sonnenberg, J. L.; Williams; Ding, F.; Lipparini, F.; Egidi, F.; Goings, J.; Peng, B.; Petrone, A.; Henderson, T.; Ranasinghe, D.; Zakrzewski, V. G.; Gao, J.; Rega, N.; Zheng, G.; Liang, W.; Hada, M.; Ehara, M.; Toyota, K.; Fukuda, R.; Hasegawa, J.; Ishida, M.; Nakajima, T.; Honda, Y.; Kitao, O.; Nakai, H.; Vreven, T.; Throssell, K.; Montgomery Jr., J. A.; Peralta, J. E.; Ogliaro, F.; Bearpark, M. J.; Heyd, J. J.; Brothers, E. N.; Kudin, K. N.; Staroverov, V. N.; Keith, T. A.; Kobayashi, R.; Normand, J.; Raghavachari, K.; Rendell, A. P.; Burant, J. C.; Iyengar, S. S.; Tomasi, J.; Cossi, M.; Millam, J. M.; Klene, M.; Adamo, C.; Cammi, R.; Ochterski, J. W.; Martin, R. L.; Morokuma, K.; Farkas, O.; Foresman, J. B.; Fox, D. J. *Gaussian 16 Rev. C.01*, Wallingford, CT, 2016.

3. Boese, A. D.; Martin, J. M. L. Development of density functionals for thermochemical kinetics. *The Journal of Chemical Physics* **2004,** *121,* 3405–3416. https://doi.org/10.1063/1.1774975
4. Ditchfield, R.; Hehre, W. J.; Pople, J. A. Self-Consistent Molecular-Orbital Methods. IX. An Extended Gaussian-Type Basis for Molecular-Orbital Studies of Organic Molecules. *The Journal of Chemical Physics* **1971,** *54,* 724–728. https://doi.org/10.1063/1.1674902
5. Plust, S. J.; Loehe, J. R.; Feher, F. J.; Benedict, J. H.; Herbrandson, H. F. Kinetics and mechanism of hydrolysis of chloro-1,3,5-triazines. Atrazine. *J. Org. Chem.* **1981,** *46,* 3661–3665. https://doi.org/10.1021/jo00331a015
6. Rys, P., Diazotization of weakly basic aromatic amines: kinetics and mechanism. In *Physico-Chemical Principles of Color Chemistry*, Peters, A. T.; Freeman, H. S., Eds. Springer Netherlands: Dordrecht, 1996; pp 1–43. https://doi.org/10.1007/978-94-009-0091-2_1
7. Klapötke, T. M.; Krumm, B.; Pflüger, C. Isolation of a Moderately Stable but Sensitive Zwitterionic Diazonium Tetrazolyl-1,2,3-triazolate. *J. Org. Chem.* **2016,** *81,* 6123–6127. https://doi.org/10.1021/acs.joc.6b01098
8. Tamara, I. G.; Oleg, A. R.; Lenor, I. K. n. Diazotisation of Weakly Basic Aromatic and Heterocyclic Amines in Strongly Acid Media. *Russ. Chem. Rev.* **1983,** *52,* 440. https://doi.org/10.1070/RC1983v052n05ABEH002830
9. Galvão, T. L. P.; Rocha, I. M.; Ribeiro da Silva, M. D. M. C.; Ribeiro da Silva, M. A. V. From 2-Hydroxypyridine to 4(3H)-Pyrimidinone: Computational Study on the Control of the Tautomeric Equilibrium. *J. Phys. Chem. A* **2013,** *117,* 12668–12674. https://doi.org/10.1021/jp410004x
10. Hodgson, H. H.; Ward, E. R. 113. The decomposition reactions of sodium aryldiazoates. Part I. Replacement of the diazo- by the nitro-group in alkaline solution with sodium nitrite. *J. Chem. Soc. (Resumed)* **1948**, 556–559. https://doi.org/10.1039/JR9480000556
11. Feng, S.; Yin, P.; He, C.; Pang, S.; Shreeve, J. n. M. Tunable Dimroth rearrangement of versatile 1,2,3-triazoles towards high-performance energetic materials. *J. Mater. Chem. A* **2021,** *9,* 12291–12298. https://doi.org/10.1039/D1TA00109D
